# Activities of Energy and Carbohydrate Metabolism Enzymes in Rainbow Trout *Оncorhynchus mykiss* Walb. upon Introduction of 24-hour Lighting in Aquaculture in Southern Russia

**DOI:** 10.1134/S0012496624600441

**Published:** 2024-11-11

**Authors:** M. A. Rodin, M. V. Kuznetsova, M. Yu. Krupnova, A. E. Kuritsyn, N. N. Nemova

**Affiliations:** Institute of Biology, Karelian Research Center, Russian Academy of Sciences, Petrozavodsk, Russia

**Keywords:** rainbow trout, energy metabolism, enzyme activity, photoperiod

## Abstract

Activities of energy and carbohydrate metabolism enzymes (cytochrome *c* oxidase (COX), pyruvate kinase (PK), glucose 6-phosphate dehydrogenase (G6PD), glycerol 1-phosphate dehydrogenase (G1PDH), lactate dehydrogenase (LDH), and aldolase) were studied in rainbow trout *Oncorhynchus mykiss* Walb. fish grown in aquaculture in North Ossetia–Alania after introducing a regime with 24-h lighting and night feeding. COX and PK activities in the liver of fish from the experimental group were found to be significantly higher than in control fish, indicating an increase in aerobic ATP synthesis. Aldolase activity in organs of fish grown with 24-h lighting was lower than in the control fish, indicating a decrease in carbohydrate utilization in glycolysis in muscles and a lower intensity of gluconeogenesis in the liver. The differences made it possible to assume that the introduction of 24-h lighting and night feeding changed energy and carbohydrate metabolism to facilitate biosynthetic processes and, therefore, weight gain in fish.

## INTRODUCTION

A photoperiod (a proportion of light and dark hours in 24 h) affects certain fish productivity parameters in aquaculture and initiates certain changes in fish growth, nutrition, and reproductive function [1]. Experimental findings [2] have given grounds to assume that the effect of an extended artificial photoperiod on fish growth parameters is associated with a redistribution of available energy, which is redirected from gonadal development to somatic growth. Methods to increase the growth rate in salmonids by extending the light day are therefore used in aquaculture facilities (mostly outside Russia, in countries of the Northern Hemisphere) [2–4]. The effects of the photoperiod on physiological parameters in fish have been considered in the majority of relevant studies, while insufficient information is available concerning the biochemical mechanisms that regulate metabolism during light-stimulated growth. The photoperiod affects the growth regulatory processes to change the expression levels of genes for regulatory and structural proteins, which determine the metabolic and synthesis rates of structural and storage substances. Energy metabolism is an important parameter and can be used to evaluate the state for individual fish as well as fish populations. High-energy biosynthetic processes sustain the intense growth of fish, especially in their early ontogeny and first years of life [5]. We have previously studied the effect of the photoperiod in salmon juveniles artificially farmed in conditions of northern latitudes (a White Sea region). Continuous illumination (light/dark 24 : 0) has been shown to accelerate the fish growth, and changes in aerobic and anaerobic metabolism in muscles have been observed to accompany the increase in growth rate [6].

Climatic conditions of southern Russia allow trout farming at water temperatures ranging 8–18°C without lower-temperature winter periods; i.e., all-year-round feeding and grow is possible for fish. Additional lighting introduced in fisheries of southern Russia would increase the trout growth rate, to improve the efficiency of food utilization, and to reduce the time it takes for fish to reach a commercially necessary body weight. Based on the above assumption, an experiment was performed to study the effect of continuous lighting on the growth and development of trout youth (0+) in aquaculture in southern Russia (Republic of North Ossetia–Alania). The objective of this work was to determine how activities of energy and carbohydrate metabolism enzymes in muscles and the liver change in trout youth during growth and development when additional lighting and night feeding are introduced in the raising regimen.

## MATERIALS AND METHODS

Two-year-old (1+) rainbow trout fish were studied at the “Ostrov akvakul’tura” fish farm (Republic of North Ossetia–Alania). The effect of 24-h lighting on the fish growth was evaluated in the study. Two groups of trout juveniles (a mean body weight of 500 g) were formed in early September. A control group was raised with natural illumination and fed every 2 h during daylight hours; a test group was exposed to continuous lighting (light/dark 24 : 0) and fed every 2 h at night. Tanks exposed to continuous lighting were equipped with LEDs (36 W, 6500 K), which were installed at the walls along the entire perimeter. The light intensity was 700–800 lx under each LED. Additional illumination was turned on at nightfall. The natural light intensity was 10 000–12 000 lx in September and October, 6500 lx in November, and 500 lx in cloudy weather. The commercial feed Scretting Nutra HP (Italy) was used in the experiment. Fish were sampled in early September (a starting data collection day), early October, and early November; each sample included 10 fish. White muscle pieces and the liver were fixed in liquid nitrogen. Fish with a medium body weight and a medium length were collected for the study (Table 1).

**Table 1.** Mean body weight (g) and length (cm) of trout juveniles

Fish group	September 9		October 9		November 10	
	weight	length	weight	length	weight	length
Control	507.9 ± 47.9	33.0 ± 0.7	836.4 ± 38.6	38.4 ± 0.8	1370.0 ± 74.4	43.5 ± 1.0
Test	572.5 ± 28.1	35.0 ± 0.4	895.0 ± 38.8	39.6 ± 0.6	1335.0 ± 61.4	44.2 ± 0.9

Activities of energy and carbohydrate metabolism enzymes in muscles (cytochrome *c* oxidase (COX), EC 1.9.З.1; lactate dehydrogenase (LDH), EC 1.1.1.27; and aldolase, EC 4.1.2.13) and the liver (COX; LDH; pyruvate kinase (PK), EC 2.7.1.40; glucose 6-phosphate dehydrogenase (G6PD), EC 1.1.1.49; glycerol 1-phosphate dehydrogenase (G1PDH), EC 1.1.1.8; and aldolase) were assayed in each individual fish spectrophotometrically. Measurements were carried out using a CLARIOSTAR microplate reader (BMG Labtech) according to standard protocols [7–10]. Enzymatic activities were expressed in terms of µmol of the substrate (product)/min/mg protein. Protein concentrations were measured using the Bradford assay [11]. Statistical analyses were performed using common variation statistics methods, including the Shapiro–Wilk test, the Kruskal–Wallis test, and subsequent sample comparisons by the Mann–Whitney test. Results were considered significant at *p* < 0.05. Results were presented as M ± SE. The study was carried out using equipment of the Collective Use Center of the Karelian Research Center.

## RESULTS AND DISCUSSION

Our study showed that rainbow trout youth raised in tanks of the fishery in North Ossetia–Alania fed intensely at night and displayed good growth and survival parameters. The specific growth rate in the test group with night feeding was higher than in the control group (10.6 g/day vs. 9.03 g/day in the first month and 11.9 g/day vs. 10.2 g/day in the second month, *p* < 0.05).

**Fig. 1.**
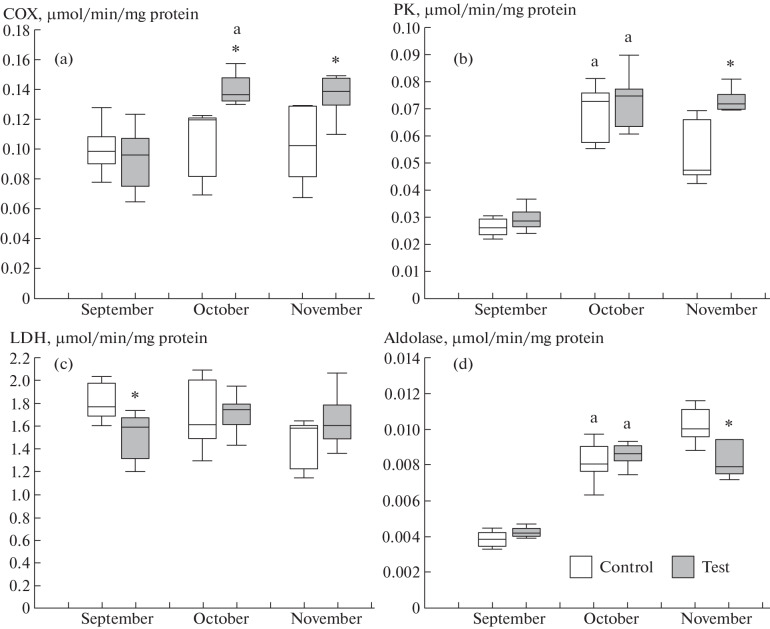
Relative activities (µmol/min/mg protein) of enzymes in the liver in rainbow trout juveniles (1+) of the control (natural illumination and daytime feeding) and test (continuous lighting and nighttime feeding) groups. Enzymes: (a) COX, (b) PK, (c) LDH, and (d) aldolase. Differences were significant at *p* < 0.05 in comparisons (*) between the control and test groups and (^a^) between the given and previous months in the same group.

Differences in enzyme activities in the liver were observed between the groups with natural illumination and continuous lighting (Fig. 1). Activity of COX, which is involved in the mitochondrial respiratory chain, was used as a parameter of aerobic metabolism [12]. COX activity in the group with continuous lighting increased in the first study month and remained higher than in the group with natural illumination during the further study period (Fig. 1a). The finding indicates that aerobic metabolism in the liver was higher in fish of the test group. Conditions created by introducing additional lighting and night feeding probably exert a favorable effect on the fish state and nutrient assimilation, in particular, because the temperature is lower and the oxygen level more stable than in the daytime. There are data that a high level of aerobic metabolism allows fish to utilize energy not only to sustain basic substance turnover and physical activity, but also to ensure highly ATP-demanding biosyntheses of structural and storage compounds [13]. PK activity in the liver in fish of the test group was higher than in the control group in November (Fig. 1b). PK activity is possible to use to characterize the production rate of pyruvate, which is utilized in aerobic ATP synthesis and acts as a precursor in fatty acid synthesis [13]. The finding, along with higher COX activity, suggests a high intensity of pyruvate production and utilization in aerobic ATP synthesis. Metón et al. [13] have shown that PK activity in the liver reflects the nutrition level and, in particular, decreases during fish starvation. Night lighting possibly acts as a factor that stimulates feeding activity. An increase in light day is known to increase the growth hormone (somatotropin) level in Atlantic salmon [14], thus stimulating both swimming and feeding activity in fish.

A redistribution of substrates utilized in energy metabolism was observed after introducing continuous illumination and changing from daytime to nighttime feeding. Aldolase activity in the liver was found to differ between the groups in November (Fig. 1d). Aldolase catalyzes the formation of dihydroxyacetone phosphate and glyceraldehyde 3-phosphate, which are then involved in glycolysis, gluconeogenesis, and lipid syntheses. Aldolase activity was lower in the group exposed to continuous lighting, suggesting a lower level and lower intensity of gluconeogenesis in the liver [15].

Activities of G1PDH and G6PD in the liver did not differ between the trout groups. G6PD is a key enzyme of the pentose phosphate pathway, which produces pentoses and generates NADPH, which acts as a reducing agent and is utilized in fatty acid and cholesterol biosyntheses [16]. The role that G1PDH plays in the liver is mostly related to glycerol 1-phosphate production from carbohydrates, and glycerol 1-phosphate is utilized to synthesize both structural and storage lipids [17, 18]. Thus, the processes that couple the glucose decomposition pathways with biosynthetic pathways were not affected by overnight lighting and the change in feeding regimen.

Based on the kinetics of changes in enzyme activities in the liver, PK, aldolase, and G6PD activities increased in both test and control groups after 1 month of the experiment (Figs. 1b–1d). The finding indicates that carbohydrate utilization in glycolysis and the pentose phosphate pathway increased, as is necessary for biosynthetic processes during trout growth. 

**Fig. 2.**
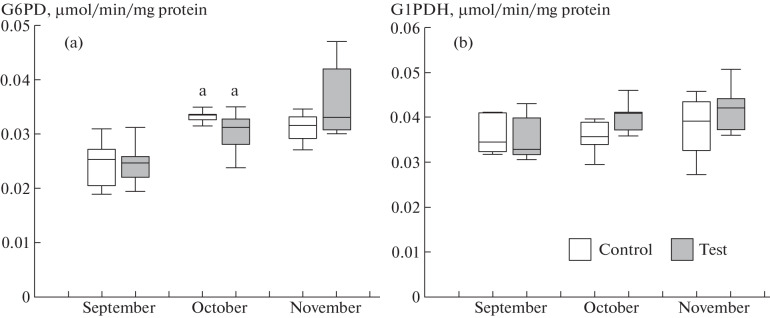
Relative activities (µmol/min/mg protein) of enzymes in the liver in rainbow trout juveniles (1+) of the control (natural illumination and daytime feeding) and test (continuous lighting and nighttime feeding) groups. Enzymes: (a) G6PD, (b) G1PDH. (^a^) Differences were significant at *p* < 0.05 in comparisons between the given and previous months in the same group.

**Fig. 3.**
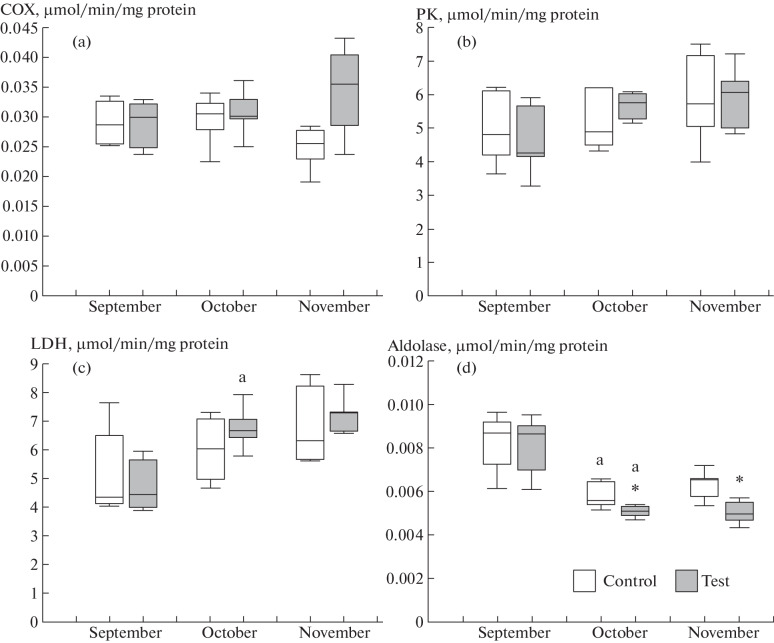
Relative activities (µmol/min/mg protein) of enzymes in white muscles in rainbow trout juveniles (1+) of the control (natural illumination and daytime feeding) and test (continuous lighting and nighttime feeding) groups. Enzymes: (a) COX, (b) PK, (c) LDH, and (d) aldolase. Differences were significant at *p* < 0.05 in comparisons (*) between the control and test groups and (^a^) between the given and previous months in the same group.

In muscles, COX activity did not differ between the groups of rainbow trout juveniles (Fig. 3a). COX activity decreased in the group with natural illumination in November, but remained stable in the group exposed to continuous lighting (Fig. 3a). The finding possibly indicates that the level of aerobic metabolism was maintained stable in the test group because light affected feeding activity and the efficiency of nutrient assimilation [1, 19].

Aldolase activity was found to differ between the groups in October and November (Fig. 3d). Lower aldolase activity was observed in the group exposed to continuous lighting, suggesting a shift from carbohydrate utilization to utilization of other substrates in supplying energy to muscles utilization [15].

The effects of additional lighting and nighttime feeding on the rainbow trout state agree with data from our previous study, which has been performed in Atlantic salmon youth raised in a fishery of North Ossetia–Alania [20]. Our experiment has shown that aldolase activity in the liver and muscles was lower and that PK activity in the liver was higher in salmon yearlings grown with continuous lighting and fed round the clock. Certain regularities are possible to assume for metabolic rearrangements that occur after the introduction of continuous lighting. Namely, a redistribution of substrates utilized in energy metabolism takes place.

Thus, our experimental data indicate that the aquaculture regimen with continuous lighting and nighttime feeding (when the temperature is lower and the oxygen level remains stable) exerts a favorable effect, facilitating better assimilation of nutrients and a higher weight gain in rainbow trout juveniles. The changes are due to rearrangements in energy and carbohydrate metabolism. The results can be used to support the introduction of continuous lighting and nighttime feeding in rainbow trout raising protocols utilized in ecological conditions of North Ossetia. The introduction may increase the weight gain in fish and, therefore, the commercial yield of the farm.
